# Clinical Holistic Medicine: Holistic Pelvic Examination and Holistic Treatment of Infertility

**DOI:** 10.1100/tsw.2004.14

**Published:** 2004-03-04

**Authors:** Søren Ventegodt, Mohammed Morad, Joav Merrick

**Affiliations:** ^1^ The Quality of Life Research Center, Teglgårdstræde 4-8, DK-1452 Copenhagen K, Denmark; ^2^ Division of Community Health, Ben Gurion University, Beer-Sheva, Israel; ^3^ National Institute of Child Health and Human Development, Office of the Medical Director, Division for Mental Retardation, Ministry of Social Affairs, Jerusalem and Zusman Child Development Center, Division of Pediatrics and Community Health, Ben Gurion University, Beer-Sheva, Israel

**Keywords:** quality of life, QOL, human development, holistic medicine, public health, holistic health, pelvic examination, child sexual abuse, infertility, Denmark, Israel

## Abstract

In clinical holistic practice, it is recommended that ample time is spent with the gynecological or pelvic examination, especially in cases of women with suspected old emotional traumas following early childhood cases of incest or sexual abuse. The holistic principles of holding and processing should be followed with the purpose of healing the patient, re-establishing the natural relationship with the body, sexuality, and reproductive organs. Sexual violations are often forcibly repressed. It appears that the tissues that were touched during the violation often bear the trauma. It is characteristic of these patients that their love lives are often problematic and do not provide the necessary support to heal the old wounds in the soul and therapy is therefore indicated. When this is concerned with the reproductive organs, it poses particular difficulties, as the therapy can easily be experienced as a repetition of the original violation, not least due to the risk of projection and transference. There is, therefore, a need for a procedure that is familiar to and safe for the patient, for all work that involves therapeutic touching of sexual organs over and beyond what is standard medical practice. This paper presents one case story of earlier child sexual abuse and one case of temporary infertility. We have established a procedure of slow or extended pelvic examination, where time is spent to make the patient familiar with the examination and accept the whole procedure, before the treatment is initiated. The procedure is carried out with a nurse, and 3 h are set aside. It includes conversation on the present condition and symptoms; concept of boundaries; about how earlier assaults can be projected into the present; establishment of the therapeutic room as a safe place; exercises on when to say “stop ”; therapeutic touch; visualization of the pelvic examination step by step beforehand; touching on the outside of the clothes with repetition of the “stop ” procedure if necessary; pelvic examination paying special attention to traumatized (damaged/scarred/blocked) areas with feel, acknowledge, and let go of the traumatized areas; postprocessing of emotions and traumas with final healing. The patient cannot be healed until negative decisions are found and dropped with a tour back to the present, to let go of negative sentences and ideas, and a plan for further positive progress.

## INTRODUCTION

The pelvic examination is a common examination performed in general practice. Whenever a woman complains of pain in the abdomen, the general practitioner is, in principle, obliged to carry out a pelvic examination in order to rule out ectopic pregnancy, acute inflammation of the lower abdomen, or something else that can seriously affect the patient. The patient is examined in the traditional gynecological position with her legs in stirrups, after which the physician can inspect, examine, explore, and take samples.

When we speak to women about their experiences in this situation, a surprisingly large number of women report that they have felt humiliated and devalued by the procedure that is normally followed. They often find it insulting to be put in positions in which their reproductive organs are exposed, without being in any way able to look after themselves. They often feel incapable of protecting themselves against an examination that may be insensitive, rushed, and does not leave space for them as human beings, the sole intention being to perform a physical procedure as efficiently as possible.

When there is an actual sexual trauma, the situation is even more complex. The purpose is the healing of the patient, to re-establish the natural relationship with the body, sexuality, and reproductive organs. Also it is important that patients acknowledge the suspected sexual violations. For integration of presumed traumas following incest and sexual assaults, it is recommended that a slow pelvic examination be carried out, based on the holistic principles of holding and processing. On top of the normal examination in such cases, all the legal aspects according to the law in the specific country must also be followed.

Many gynecological problems like involuntary childlessness or infertility seem to follow problems in the woman's relationship with her body, gender, and sexuality, which might be alleviated by a holistic approach to the woman and the gynecological procedures. It is important that the woman experiences being seen and acknowledged as a whole person, where she feels herself and all parts of her body deeply accepted. This approach can change the often quite provocative pelvic examination from a fearful to a peaceful or even healing experience. Sometimes a few hours of work can change the woman's perception of herself, her body, her gender, and her sexuality, but this is usually not done in a busy general practice.

## CLINICAL HOLISTIC MEDICINE IS BASED ON THE LIFE MISSION THEORY AND THE HOLISTIC PROCESS THEORY OF HEALING

The life mission theory[1,2,3,4,5,6] grants that everybody has a purpose in life or a talent. Happiness comes from living out this purpose and succeeding in expressing the core talent. To do this, it is important to develop as a person into what is known as the natural condition, a condition where the person knows himself and uses all his efforts on achieving what is most important for him. The holistic process theory of healing[7,8,9,10] and the related quality of life theories[11,12,13] state that the return to the natural state of being is possible whenever the person gets the resources needed for existential healing. The resources needed are, according to the theory, awareness, respect, care, acknowledgment, and acceptance with support and processing in feeling, understanding, and letting go of negative attitudes and beliefs. The precondition for holistic healing to take place is trust with the intention that the healing *will* take place. Existential healing is not a local healing of any tissue, but a healing of the wholeness of the person, making him much more resourceful, loving, and knowledgeable of himself, his own needs, and wishes. To let go of negative attitudes and beliefs, the person returns to a more responsible existential position with an improved quality of life (QOL). The philosophical change of the person in healing is often a change towards preferring difficult problems and challenges, instead of avoiding difficulties in life[14,15,16,17,18,19,20,21]. The person who becomes happier and more resourceful often also becomes more healthy, more talented, and more able to function[22,23,24].

## THE PELVIC EXAMINATION

It is worrisome that the numerous physicians and gynecologists around the world subject women to examinations that in themselves may be stressful and perhaps even traumatic experiences. Part of the problem is obviously due to shortage of time, but another important part of the problem appears to be due to misunderstood respect for the woman's sexual boundaries, which means that the physician feels more secure in reducing the female patient to a pure object of examination. It is considerably easier to examine a set of organs than to relate to a living person, with feelings of shame and desire and a sexuality that can threaten to end the career of the physician, if he so much as relates to it.

Mere suspicion that the physician may assault the woman, who is placed in what is a very vulnerable position, can cause the physician to entrench himself behind this clinical facade in a way that is in itself dehumanizing. Instead of being present, the physician almost tries to avoid being there, and becomes an excuse for himself. Paradoxically, this gives rise to another type of violation — being rummaged around in the woman’s most delicate parts, as though one was something rather like a car engine. Pushed to its extreme, it is as though the medical profession has decided once and for all that it is difficult to show human respect and care in the situation where the patient's reproductive organs are exposed. Instead, it is necessary to make do with showing the craftsman’s respect that a skilled clockmaker displays with a sophisticated timepiece.

We also have faced ethical problems when putting women or young girls who have previously been subjected to sexual assault through the general examination procedure, because this procedure can brings back memories of the assault. It is not possible to solve a problem of that kind by simply passing the buck on to the gynecologist who, although he has more experience, generally has far less knowledge of the patient.

One of the emotionally most difficult aspects of the pelvic examination is the physical touch itself, which the gynecologist tries to make less dangerous by using rubber gloves and instruments. Due to a strict professionalism with often a brusque silence (because the physician is afraid of saying the wrong thing), the women can sometimes be reminded of up-tightness, bad sexual experiences with insensitive lovers, or even insulting sexual touches, rough partners, attempted rape, or in the worst case assault in childhood.

Where sexually harmless situations are concerned, the physician generally does not have any objection to calming the patient through touch, for example by putting his hand on the arm of a woman who is upset. This often causes difficulties in the gynecological context, because if the situation is misunderstood by the woman, the entire medical career of the physician can be finished in an afternoon. It is important that both the patient and the physician realize that instead of avoiding any human touch in connection with a pelvic examination, the physical touch can and must also be an entirely natural constituent element here too. As in any other emotionally difficult situation, supporting physical touch may help the woman to feel acceptance and support, and in that way promote her sense of security in the situation and not least her confidence in the physician and the treatment.

Many male medical students at first have serious problems with the pelvic examination (as an example, one student became impotent for months after spending a period of time in a gynecology department). We also often encounter patients who clearly hated the pelvic examination because it reminded them of unpleasant things from their past. There we must consider whether it might not be possible to turn the unavoidable touching of the woman around, so that it becomes not an evil that has to be minimized, but a therapeutic resource that can be drawn on or, in other words, instead of masking the touch, using it to express respect for and care of the woman in the examination situation.

## THE HOLISTIC PELVIC EXAMINATION

Due to the fact that we had a patient with a need for a considerate procedure of this type, we arranged for a very thorough, careful, and well-planned procedure, which specifically tried to avoid turning the patient into an object and instead to treat the woman as a woman. The idea was to make the pelvic examination slow — very slow, in fact, so slow that the physician could be sure that the patient was entirely there at all stages of the examination, indeed in everything that was done with her. We also went through the procedure with the nurse, who approved it. We found, to our surprise, that the pelvic examination was in fact healing and therapeutic for the patient when it was performed in this slow and attentive way. The physician (SV) also discovered that it was not unpleasant to be present in the examination situation. The new and more relaxed attitude and new acceptance of this unavoidable physical touching of the woman's reproductive organs led to a surprising change in the patient's experience of the examination.

With this new approach, women started to say that it was nowhere near as bad as it used to be. In contrast to what might have been imagined, the empathic and physically present form of examination also becomes less sexually provocative for the physician than the normal, rapid gynecological procedure. Since that time, we have allowed ourselves an extra amount of time when we have had female patients with sexual problems who perhaps have been subjected to sexual assault — the truth of which, however, it is never possible to know for sure — but who have been very vulnerable, sensitive, and perhaps even full of shame and self-condemnatory in relation to their sex, reproductive organs, and sexuality.

If sexual assault is suspected, we use the slow procedure with preparation of the patient, where she is thoroughly informed about it beforehand, so that we can be sure of her complete acceptance and assistance throughout the procedure.

In the medical literature, it is normally recommended that a nurse or other person should always be present when the physician performs gynecological or sexological procedures. This is not for the sake of the patient, as some people perhaps think, but to protect the physician, because this way there is a witness, if a patient one day wishes to lodge a complaint about sexual assault. In Denmark, where it is extremely rare for cases to come to court, there are many general practitioners who never have the nurse with them for a pelvic examination and the physician gradually adopts a natural and relaxed attitude to this. Today in our holistic practice, the nurse is brought in if the patient needs extra holding, but the nurse is not brought in if an extra person would disrupt the examination. A case history of sexual assault follows below.

### A Case Story

Female, aged 28 years, with pain in lower abdomen, sexual problems, and suspicion of incest is seen for the first quality-of-life (QOL) conversation. On the couch, we work (through conversation) on her 12 years of problems with her failure and loss of confidence. She still has pain in the right side of the lower abdomen and a pelvic examination should be performed, but in view of the delicate mental state of the patient, it is deferred.

Second QOL conversation: Talked about boundaries — being below the line in relationship with her father, above it, having completely disappeared and taking up the whole space = taking responsibility for everything. Exercise for next conversation: Find and describe situations where there are good examples of this.

Third QOL conversation: She had problems in her anus, which tore, and she felt a large cavity in her abdomen together with constant problems in the lowest part of the large bowel. She suspects, with horror, sexual assaults in the anus, but cannot remember anything. “It would fit in well with what I feel,” she said. She related that her first intercourse broke her hymen. There was no way of knowing what trauma lied within her and they will not emerge until she has had enough confidence to accept the holding. She has had desire for her boyfriend three times, but the last time she had to stop, as the desire did not last.

Fourth QOL conversation: We talked about her anxiety about coming here, about going into therapy, about meeting me (SV) as a man, about the anxiety, which she suffers from frequently. We talked about philosophy of life. We talked about access to feelings, sex, and love by noting what is hidden in the body. Exercise for next conversation: Feel all the emotions you have. Stop and feel them. Write down what you feel on a piece of paper.

Fifth QOL conversation: Things have gone well, she said. “What did you think about on the way to see me?” “It’s a bit like school — as though I have to perform well,” she said. “I find the situation with you today very stiff,” I answered her. We talked about this. She has felt alone, avoided trust and closeness. She has done her homework: She would like to have desire for her boyfriend, but does not, just hopes he will not come and take her. We talk about this. While she is on the couch, we work through the conversation to get into her feelings. It becomes very sensitive, and it appeared as though she was about to be suffocated in the gestalt and feelings that came to the surface. She cried and said “It does not matter to me” and “I do not care”.

Sixth QOL conversation: On the couch, we work on being present in the body, she lies on her back with her legs spread out and feels hard pressure across her chest and lower abdomen; I support her on the thigh and across the top of the head. She cried silent therapeutic tears and afterwards she was better. No exercise for next time, it is going the way it should without.

Seventh QOL conversation: Wanted to get to the couch straight away and does so. We talked about taming her like the little prince — she understands that well. We work on tensions in the low back, lower abdomen, pelvis, and thighs. The sartorius muscle [which runs across the thigh] in particular is extremely tense and is very sore when she spreads her legs. We talk about her being chronically tense to close her lap and hide her sex. She was also very sore in the left knee “because I hide myself [i.e., her reproductive organs] by pushing my pelvis backwards”. Exercise: Stop and feel, when you feel something. Allow space for your emotions. They are your life energy, regardless how difficult they are.

Eighth QOL conversation: … She cries a lot and does not think anything is happening at all. She has come to a standstill. On the couch, we work on her abdomen; she is still sore, corresponding to the large bowel on the left side. Pelvic examination still indicated, but not urgent, since it is difficult to obtain permission to approach the regions without her having the sensation of being assaulted and this must be respected. We talked about the paradox: the more she goes into the gestalt, where she has emotionally “died”, the more she feels she is not getting anywhere, but that is a good sign therapeutically. Exercise for next week: Accept that you have come to a standstill. Spend time being at this standstill. Do not force yourself to do anything. Just be at a standstill.

Ninth QOL conversation: Has been very, very far away and sad. On the couch, we continued to work on joining her two halves above and below the navel. It is as though she runs away from feeling everything below the navel. Being together with her boyfriend has returned the trauma with focus in the area of her lower abdomen and she felt suffocated. She needs to do the same exercise as homework, since she had not done it for this time. Pelvic examination still indicated. We agree that next time there will be a long session with our nurse, where we perform an extended pelvic examination with respect to identifying traumas in the tissue. We make it by establishing a safe place or point. The whole procedure is visualized. Everything takes place very slowly, and after practicing the stopping procedure. We are to talk about projections of the assault into the present. A plan is drawn up, with which the patient associates herself fully before we start; 3 h are set aside.

Tenth QOL conversation and session with the nurse: We run through the case report together and it adds up to suspicion of sexual assault. Following acceptance by the patient, we implement a slow pelvic examination for integration of presumed traumas following incest and sexual assaults. The purpose is re-establishment of the natural relationship with the body, sexuality, and reproductive organs in the patient, who has problems due to acknowledged or suspected sexual violations.

Sexual violations are often forcibly repressed. It appears that the tissues that are touched during the violation often bear the trauma. It is characteristic of these patients that their love lives are often problematic and do not provide the necessary support to heal the old wounds in the soul, and therapy is therefore indicated. When this is concerned with the reproductive organs, it poses particular difficulties as the therapy can easily be experienced as a repetition of the original violation, not least due to the risk of projection and transference. There is, therefore, a need for a procedure that is familiar and safe for the patient, but it involves therapeutic touching of sexual organs over and beyond what is standard medical practice. We establish the following procedure, with which the patient is familiarized and accepts, before the treatment is initiated. The procedure is carried out with a nurse and ample time allocated (3 h). The procedure includes:

- Conversation about the present condition — relationship to body, sexuality, and reproductive organs, including investigation of problems in sex life such as pain and painful memories are recapitulated.
- Conversation about the concept of boundaries, so that the patient understands fully where her own sexual boundaries are in order that the examination is not experienced as an assault.
- Conversation about how the assault can be projected into the present. How do the patient and therapist act if the patient finds the therapy a violation? It is important to say so immediately if something feels unpleasant or wrong.
- The establishment of the therapeutic room as a safe place.
- Stopping exercise — touching of the body and reproductive organs on the outside of the clothes, where the patient says stop and the hands are removed at once.
- Contact: Physical touching of the body — from the head down to the stomach, pelvis, and lower abdomen, slowly and in suitable steps, so that the patient is present and secure throughout.
- Visualization of extended pelvic examination, where the therapist runs through the steps of the examination thoroughly, so the patient can imagine them before they are due to happen.
- Touching on the outside of the clothes with repetition of the “stop” procedure if necessary.
- Pelvic examination paying special attention to traumatized (damaged/scarred/blocked) areas.
- Feel, acknowledge, and let go of the traumatized areas. If there are areas that appear blocked or “the patient not present”, has pains, or other discomfort, we then give special attention with regard to their integration. This is not fundamentally different, for example, from the treatment of growing pains in children by touching the areas that are sore, such as around the knee. If the sick areas are attended, they are also usually healed.
- Postprocessing of emotions and traumas. The work with blocked places in the body often releases painful gestalts from childhood and adolescence, which must be talked through in the same way that the patient’s painful feelings must be supported and accommodated by both physician and patient.
- Healing is only possible when negative decisions are found and dropped. The patient has to come back to the present, let go of negative sentences or ideas, and plan for further positive progress.
- These points above are printed out, signed, and approved by the patient as a formal contract.

This procedure was carried out with success in this patient. The gynecological and rectal examination was normal except for a lesion of blockage type ($3 \times 1$ cm in size) with brownish, folded skin between labium minor and labium major on the left side (which is processed with partial success) and tenderness in the vagina 5 cm up on both sides, but nothing abnormal discovered. There were problems with the “stop and start” safety procedure, which we had to repeat 20 times in 4 series before she could say yes and no. She was happy and at ease after the procedure, but a little disappointed at not having broken down/having achieved a breakthrough.

Eleventh QOL conversation: She has felt lighter and more free since last time. She has not menstruated, but pregnancy test is negative. She must test again in 3 weeks if menstruation does not arrive. She feels pressure over her lower back, as though it is on the way, but it does not come. This is a normal body reaction and she has to be calm, but remember that the most common reason for a period not to occur is pregnancy in her age. She has started to say stop, in relation to her boyfriend sexually, when she does not feel desire. She relates that he respects this, but then she herself has great desire. She needs to say no sometimes during intercourse and we discuss how they must practice this together.

This conversation was a very moving experience for the three people present (the patient, the physician, and nurse). The patient was shaking with nervousness when she arrived, but quickly adapted as she got on very well with our nurse. It was clear that the patient found it very difficult to say no or stop to others. In our preparatory training procedure, she had to say stop about 100 times before she could continue the session. For us as physicians, it is hard to comprehend how someone can become an adult without such a fundamental skill because she will constantly be in conflict in relation to men or sex, since she is not able to set boundaries.

So, although the patient felt disappointed that she had not “broken through” after the extended pelvic examination, in fact she had. This treatment became a turning point in her life and in the therapy. After that session, she began to let go of what she was clinging to and what limited her. So in fact, that session was a very important and moving session. The patient did not like the look of her external reproductive organs because of the dark and blocked area between the labia on one side. Nothing was noted if one did not look for blockages, but the area was clearly in the patient's negative focus. It was found to be very important for her sexual self-esteem to have the problems confronted here. The important aspect is that after the procedure in which the physician finds such an area of embarrassment, he is forced to touch the very place of which the patient is most ashamed. It is there that she is confronted with her own self-condemnation, which can then be processed and integrated. The nurse had a very important role as the supporter as she held the patient in her hands, while the physician carefully gave contact. It is also important to note that the patient’s concern about pelvic examination afterwards has largely disappeared.

So we do not just need attention, respect, and care — and acknowledgment of our soul — we also need something bodily, physical, and down-to-earth, namely acceptance of our sex. As a physician, when it is possible to meet the patient with respect and within boundaries to recognize her as a woman, then we can help her and give her our full acceptance. Many problems related to sexuality then appear to decrease, as they are probably due to self-condemnation and lack of sexual self-acceptance.

## INVOLUNTARY CHILDLESSNESS, INFERTILITY, AND PERSONAL DEVELOPMENT

Sometimes, when a relationship is not working, the result can be unwanted childlessness or infertility. It frequently happens that couples previously unable to conceive, have a child when one or both partners start on self-development. The physician can initiate this process of healing by a holistic approach, as shown in the following case story.

### A Case Story

Female, aged 30 years, with involuntary childlessness and the first QOL conversation. Involuntary childlessness or infertility for several years. Investigated by her gynecologist, who found everything to be normal. Social history taking revealed that her mother had disappeared when the patient was 4 years old. She had sleeping problems, which began when she found her mother again 2 years ago and afterwards died of heart disease 1 year ago. Evaluation after the conversation: The patient does not appear to be a giving person, was a neglected child with a “closed heart”, who is only able to receive, which is even a problem. We discuss responsibility for one’s own life, know yourself, your needs, and make sure that you have them fulfilled, then you will be able to have a good life. Plan for next conversation with two exercises: Make a complete list of all the problems in your life (working life, social life, family, sex life, and friends). Make a list of everything you want for yourself, of your deep, genuine needs and what would make you completely happy. What do you long for? Come back in 14 days.

Second QOL conversation: The patient broke down after the last time she came to see me and has had a hard time since. We talk about what feelings surfaced. She felt immense grief with a feeling that the world simply came to a standstill. During the weekend, she became completely hysterical, unable to understand why her friends could be able to chat about trivial matters, when an acquaintance had just died. She became the unreasonable little child sitting in the car again. She was told during the conversation that “You must not anticipate becoming pregnant for the first 3 years” to which she was shocked, but later accepted and bought a cat instead. Concerning the exercise, she came with four pages of problems and one page of wishes. Most important was her statement that “I am not satisfied with myself and not happy either”, followed by “I have not had a child yet by the age of 30 years and “I do not know what I want, I do not know my wishes”. Her most important wish was to have a child, followed by becoming happy, and able to be something for other people. Her needs were “to get to know myself — to find out what I want to do”. Exercise for next time: Come with a deeper and more true version of the same lists — so that you can fold out and go further in your growth and development, because now you seem to have come to a complete stop. New appointment scheduled in 2 months.

Third QOL conversation: Has finally become pregnant — just like that. She feels fine now. We talk about relaxing in relation to the pregnancy — taking things as they come, since everything looks fine now. She should do everything she is good at in terms of work, traveling, and experiencing the world with her own family. Has rediscovered some decisive confidence in life and found her surplus. Exercise for next meeting: Read some books (suggestions given), which will give her “food for thoughts” and ideas to think about. Come back in 2 months.

This patient is going through a painful process in which she is letting go of her compulsory need to become pregnant here and now. From a biomedical point of view, it is completely unreasonable to torment the patient and burden her with all the strenuous exercises. From a holistic perspective, this is necessary for her awareness, growth, and drive in life. Maybe there is a very often a good reason for temporary infertility, as not all women are ready to become mothers and give constant care for the next 12–18 years, which children need them to do. Nature is wise and very often it is better to rely on the deep wisdom of the body, than mechanically to force a solution. If the woman is not ready to have a child, it is hardly good for the child to be born now either.

It appears as though people who wrestle with their existential problems find it more difficult to have children than people who do not. It is as though body and soul know well that now is not the time to have children. The unfortunate aspect is that these same people in their daily life do not have access to this deep wisdom.

## DISCUSSION

It is important to notice that we introduced a slow pelvic examination with a therapeutic element, relevant for a wide range of psychosomatic disturbances related to gender and sexuality, from infertility to gynecological and sexual psychosomatic problems and the long-term consequences of child sexual abuse. On one hand, for a clinical practice this opens up many beneficial and healing qualities for the patient, because it allows a much closer and more intimate relationship between the patient and the physician than has been the traditional practice, but on the other hand this procedure has several disadvantages.

In many cultures, this cannot be practiced due to cultural or religious reasons and a strong sexual taboo, that the female will experience the process as overwhelming or even insulting. In the U.S., it might be practically impossible to follow our recommendation in many cases because of the time consumption, economics, and reimbursement issues of this culture and the heavy “malpractice culture” in that country.

The most difficult problem of this procedure seems to be that is makes it very difficult to be sure that the procedure and all the involved steps are always necessary and rational. This procedure and the cultural issues involved means that it has a high potential for malpractice, but this can be minimized dramatically by the following steps: (1) before the procedure is done, the patient must read about it with at least one case study, like the one in this paper, to fully understand the emotional and existential implications of the procedure, so she has time to contemplate and make her decision of whether or not to accept the physicians offer; (2) the procedure is also orally presented by the physician to the patient before she signs the contract; (3) the physician must be in supervision to discuss the problems if any about borders, intimacy, emotional, and sexual issues. Close supervision and full intercollegial openness is the best prevention of malpractice, as malpractice often occurs with physicians without a network and without openness about what is going on in their clinic.

## CONCLUSION

The holistic pelvic examination is designed for solving gynecological and sexual problems of psychosomatic origin. It is a recommended alternative to the standard procedure whenever there is a suspicion of a history of sexual assault or sexual abuse, even when that abuse took place many years ago. It is often more time consuming and can involve strong emotions on the part of the patient, as earlier unresolved traumas are contacted during the examination. In the holistic pelvic examination this is not a problem, but quite opposite the release of suppressed emotions might be healing to the patient, if the physician knows how “to hold” (meaning to care for) the patient and how to process the problems and emotions in order for the patient to heal.

A holistic approach in general can help the woman not to feel humiliated or devalued by the pelvic examination procedure, but instead respected, acknowledged, and accepted as the woman she really is. Sometimes this is all it takes to solve even more severe medical problems like involuntary childlessness, as shown in the presented case.

## ACKNOWLEDGMENTS

This study was supported by grants from IMK Almene Fond. The quality of life research was approved by the Copenhagen Scientific Ethical Committee under number (KF)V.100.2123/91.

## BIOSKETCHES

**Søren Ventegodt, MD,** is the Director of the Quality of Life Research Center in Copenhagen, Denmark. He is also responsible for a Research Clinic for Holistic Medicine in Copenhagen and is a popular speaker throughout Scandinavia. He has published numerous scientific or popular articles and a number of books on holistic medicine, quality of life, and quality of working life. His most important scientific contributions are the comprehensive SEQOL questionnaire, the very short QoL5 questionnaire, the integrated QOL theory, the holistic process theory, the life mission theory, and the Danish Quality of Life Research Survey, 1991–94 in cooperation with the University Hospital of Copenhagen and the late pediatric professor Bengt Zachau-Christiansen. E-mail: ventegodt@livskvalitet.org. Website: www.livskvalitet.org

**Mohammed Morad, MD**, is Specialist in Family Medicine, Lecturer in Family Medicine at the National Institute of Child Health and Human Development, Division of Community Health, Ben Gurion University of the Negev and the Medical Director of a large area clinic in the city of Beer-Sheva. He has publications on Bedouin health, health aspects, spiritual health, and aging in persons with intellectual disability, and is a presenter on topics such as health policy and services for the disadvantaged at national and international conferences. E-mail: morad62@barak-online.net

**Joav Merrick, MD, DMSc,** is Professor of Child Health and Human Development affiliated with the Zusman Child Development Center, Division of Pediatrics and Community Health at the Ben Gurion University, Beer-Sheva, Israel; the Medical Director of the Division for Mental Retardation, Ministry of Social Affairs, Jerusalem; and the Founder and Director of the National Institute of Child Health and Human Development. He has numerous publications in the field of child and human development, rehabilitation, intellectual disability, disability, health, welfare, abuse, advocacy, quality of life, and prevention. Dr. Merrick received the Peter Sabroe Child Award for outstanding work on behalf of Danish Children in 1985 and the International LEGO-Prize ("The Children's Nobel Prize") for an extraordinary contribution towards improvement in child welfare and well being in 1987. E-mail: jmerrick@internet-zahav.net. Website: www.nichd-israel.com
